# Fundamental Difficulties Prevent the Reconstruction of the Deep Phylogeny of Viruses

**DOI:** 10.3390/v12101130

**Published:** 2020-10-06

**Authors:** Jean-Michel Claverie

**Affiliations:** Structural & Genomic Information Laboratory (IGS, UMR 7256), Mediterranean Institute of Microbiology (FR3479), Aix-Marseille University and CNRS, 13288 Marseille, France; jean-michel.claverie@univ-amu.fr

**Keywords:** origin of viruses, phylogenetic reconstruction, reductive evolution, obligate intracellular parasites, *Varidnaviria*, *Bamfordvirae*, *Nucleocytoviricota*

## Abstract

The extension of virology beyond its traditional medical, veterinary, or agricultural applications, now called environmental virology, has shown that viruses are both the most numerous and diverse biological entities on Earth. In particular, virus isolations from unicellular eukaryotic hosts (heterotrophic and photosynthetic protozoans) revealed numerous viral types previously unexpected in terms of virion structure, gene content, or mode of replication. Complemented by large-scale metagenomic analyses, these discoveries have rekindled interest in the enigma of the origin of viruses, for which a description encompassing all their diversity remains not available. Several laboratories have repeatedly tackled the deep reconstruction of the evolutionary history of viruses, using various methods of molecular phylogeny applied to the few shared “core” genes detected in certain virus groups (e.g., the *Nucleocytoviricota*). Beyond the practical difficulties of establishing reliable homology relationships from extremely divergent sequences, I present here conceptual arguments highlighting several fundamental limitations plaguing the reconstruction of the deep evolutionary history of viruses, and even more the identification of their unique or multiple origin(s). These arguments also underline the risk of establishing premature high level viral taxonomic classifications. Those limitations are direct consequences of the random mechanisms governing the reductive/retrogressive evolution of all obligate intracellular parasites.

## 1. Introduction

Since the serendipitous discovery of the tobacco mosaic virus by Dmitri Ivanovsky in 1892 [1], virology has mostly focused on those viruses responsible for (often dreadful) diseases of human, animals, or plants. Most of the research was then dedicated to the mechanisms of pathogenicity rather than to the physiology of the viruses as biological entities [2]. One noticeable exception was the study of bacteriophages that led to the basic concepts of modern cellular biology and to many of today’s molecular biology tools [3]. About fifteen years ago, a second wind of virology occurred with the discovery of the first giant viruses infecting amoeba [4,5], rapidly followed by that of an unexpected diversity of related viruses associated with other protozoan and algal hosts [6,7,8,9,10,11]. Through isolation studies [12,13,14,15,16] complemented by large-scale environmental metagenomic explorations [17,18,19], a flurry of new viruses has since been uncovered exhibiting unexpected virion sizes and morphologies, unusual gene contents, or exotic modes of replication [12,20,21,22,23]. Beyond their unanticipated diversity, these “unconventional viruses” (not to be confused with the infectious agents now known as prions) [24,25] challenged the established borders between the viral and cellular worlds and revived interest in the study of the deep phylogeny of eukaryotic dsDNA viruses, right down to the question of their origin. However, these works are struggling to converge towards a unanimous scenario, due to the difficulty of applying the usual molecular phylogeny approaches to the extreme diversity of viral gene contents, and to the low sequence similarity exhibited by the rare “core” proteins that they have in common [12,26,27,28,29,30,31,32,33,34,35]. Beyond these practical difficulties, this article will focus on even more fundamental limitations plaguing the reconstruction of the deep evolutionary history of all viruses.

## 2. All Viruses Are “Unconventional”

All viruses are unconventional, in the sense that this category of microorganisms lacks a set of universal homologous components that they all share, and that could be used to define the “norm” that any virus should obey to be recognized as such. To clarify the significance of this unique feature of viruses, I will take the counterexample of the cellular world which encompasses an enormous diversity of cell types, from the already highly diverse prokaryotes (Archaea and Bacteria domains), to the eukaryotes. Yet, all the “conventional” cell types will share homologs of the enzymes and structural proteins required to replicate their DNA genome, to express their genes, synthetize their proteins, together with a number of metabolic pathways to synthetize amino-acids, nucleotides, and generate ATP.

There are two fundamental consequences to the existence of such common set of components and subsystems. First, except for the smallest details, what is learned on a given cell type can usually be transposed to many others. Ribosomes, for instance, work the same way in all cells. The notion of “model” systems (such as *Escherichia coli* or yeast) has thus become a founding stone of modern Biology. This is well summarized by the aphorism: “what is true for *E. coli* is true for the elephant”, attributed to Jacques Monod and Francois Jacob [36], two of the most prominent founders of molecular biology. Second, unconventional cells are then easy to distinguish from the “normal” crowd, because they lack one of these (almost) common capability: a functional cell division apparatus (e.g., *Babela massiliensis*) [37], ATP synthesis (e.g., *Rickettsia*) [38], and/or amino-acid or nucleotide synthetic pathways (e.g., *Chlamydiae* or *Tremblaya princeps*) [39,40].

If we now try to do the same exercise for viruses, i.e., identify a “model” species and then define “unconventional viruses” versus “regular ones”, one soon realizes that it is impossible. Amazingly, this is not even possible among members belonging to the same Baltimore’s classification, such as the dsDNA viruses (on which I will focus for the sake of clarity), or even within the same kingdom. For instance, Mimivirus (with a 1.2 Mb genome) [5] is by no mean a “model” or a prototype for all the dsDNA viruses included in the recently defined “*Bamfordvirae*” kingdom [41] that also includes the adenoviruses (with a 30 kb genome) [42] or a new type of viral parasite, the virophages [20] (Table 1). In other words: would a complete knowledge of the physiology/replication cycle of one of these viruses be of any help to elucidate that of the others? The answer is clearly no.

The fundamental reason a virus prototype cannot exist is because the term “virus” does not designate an “object” (alive or not, this question is still debated) of which a model can be built, but a conceptual process. What makes viruses alike is not what they are made of, but the cyclic scenario they use to reproduce themselves. In the most general terms, this scenario is as follows: transported in a molecular box, a genome of some kind (RNA or DNA) gain access to a cellular system that is used to produce more copies of itself, and package them into neo-synthetized boxes that are then released in the environment. This abstract scenario can be materially implemented in many different ways, many of which may not have been discovered yet, making environmental virology one the frontiers of the Terra Incognita of basic Biology [2,43]. Amazingly, the fundamental difficulty of formally defining viruses was already perceived by Lwoff in the early days of virology when the best he could do was to list the missing properties that precluded them to belong to the cellular world: viruses could not divide, could not synthetize ATP, and could not synthetize their proteins [44].

## 3. Viruses Display a Huge Gradation in “Absolute” Parasitism

One of the main properties that is common to all viruses is that the “active” part of their replication cycle can only happen inside a cell. They are “obligate” intracellular parasites, a property that most non-specialists believe is unique to viruses. However, we now know that this property alone is not sufficient to discriminate viruses from the cellular world, as modern microbiology revealed a fascinating underworld of “unconventional” parasitic cells that can only live within other cells (such as those already listed above) [37,38,39,40]. Yet, these obligate intracellular parasites (defective for different subsets of essential genes and metabolic pathways) manage to retain enough common macromolecular components so their classification as members of (or derived from) the cellular world (e.g., the bacterial domain) remains straightforward (e.g., by the presence of ribosomes).

At this point, it is interesting to notice that although “obligate” parasitism sounds like a qualitative character (i.e., either you are or are not an absolute parasite), it actually covers a whole gradation of dependency toward the host cell. In some cases, for instance, supplementing a culture medium with a specific metabolite was found sufficient to turn an absolute intracellular parasitic bacterium into a free-living one (e.g., *Tropheryma*) [45]. In other cases, the parasitic organism is short of achieving free-living in a rich medium by hundreds of missing genes (e.g., *Tremblaya*) [40]. Absolute parasitism could thus be quantified by the number of essential genes that a thought experiment would need to reintroduce into an absolute parasite to restore its free-living capacity.

Viruses can actually be ranked relative to each other in a similar way, from minimal viral genomes merely encoding the blueprints of their particle (i.e., less than a handful of structural proteins) [46,47], to giant viruses encoding, in addition to hundreds of their virion components, the blueprint of the transient intracellular factory used to synthetize them, as well as the regulatory elements required to hijack the systems unique to the host cell (e.g., the ribosomes) [12,20]. In that respect, the range of “absolute” parasitism covered by the eukaryotic dsDNA viruses is particularly baffling with cytoplasmic giant viruses encoding largely more than thousand proteins including complete DNA replication and transcription machineries [20], many protein translation components [48], and numerous biosynthetic pathways [11,12,19], down to nuclear polyomaviruses with 5 kb genomes encoding 5 proteins [46,47]. Such huge variations in genome sizes and gene contents are difficult to interpret in the context of a unique one-fit-all evolutionary scenario driven by a fixed set of fitness constraints. A similar variation in genome sizes (14–735 kb) is also seen among dsDNA bacteriophages [49,50]. Given that huge range of genomic complexity together with the lack of a sizable common set of conserved genes, it may seem quite unrealistic and artificial to postulate a common origin for all dsDNA viruses, even limiting ourselves to those infecting eukaryotes. Yet such a feat is periodically attempted (e.g., [29,33,51,52]). In the following sections, I present several fundamental reasons why deep reconstructions of viral phylogenies might not be tractable beyond the level of individual virus families.

## 4. First Argument in Favor of a Retrogressive Evolutionary Scenario

Even if the gene contents of viruses (including those infecting the same host) appear largely uncorrelated, there is some order in this apparent chaos. For instance, there seems to be a strict hierarchy governing the presence of encoded DNA-dependent DNA polymerases and of DNA-dependent RNA polymerases in viral genomes (Table 1). As of today, all eukaryotic dsDNA viruses encoding their own RNA polymerase, also encode a DNA polymerase. If the RNA polymerase can be absent from viruses encoding a DNA polymerase, the converse is not true. In other words, DNA polymerases are only absent from viruses also lacking a DNA-dependent RNA polymerase. Conditioning the presence of a virus-encoded transcription apparatus to that of a replication apparatus strongly suggests an irreversible reductive evolutionary process with a progressive loss of functions from ancestors equipped with both machineries. This is one of the arguments in favor of a cell-like origin of dsDNA viruses, including those from the newly defined *Bamfordvirae* kingdom (that include a whole spectrum of virus families with and without virus-encoded DNA/RNA polymerases) (Table 1). Further supporting such a progressive loss-of-function scenario, two intermediate virus groups (*Coccolithovirus* and the *Marseilleviridae*) encode a DNA-dependent RNA polymerase that, most surprisingly, is not packaged in their particles, forcing them to initiate their cytoplasmic replication cycle by first recruiting nuclear functions [23,53].

However, the reason why the loss of the RNA polymerase should always precede that of the DNA polymerase is not clear, as the absence of any of these genes will force a previously cytoplasmic virus to become dependent of cellular functions located in the nucleus. Once evolved to gain access to the nucleus, a virus devoid of its own DNA polymerase could use that of the cellular host, independently of the presence/absence of its own RNA polymerase. Thus, no basic biological rule would be violated by the eventual discovery of such “unconventional” dsDNA virus. Interestingly, the presence of a DNA-dependent RNA polymerase strictly conditioned to that of a DNA polymerase is also observed in all known dsDNA bacteriophages, which suggests that it is not linked to the presence of a nucleus.

Finally, another “unconventional” type of large dsDNA virus is represented by the polydnaviruses of parasitic wasps. Amazingly, if their particles could package up to 800 kb of DNA, it does not contain any of the genes required for its replication or the production of virions (reviewed in [54]). In this extreme case, even the minimal blueprint of the viral particle has been subcontracted to the host cell. It is difficult to interpret the emergence of such a virus other than as the end-point of a reductive evolution. Clearly, the fact that some viruses do not even encode the constituents of their own particles (yet a feature that does not contradict Lwoff’s formal criteria) does not help in designing a rigorous definition that will include them all.

## 5. The Main Conceptual Difficulty Plaguing the Deep Phylogenetic Reconstruction of Viruses’ Evolution

Three main scenarios have been proposed to explain the origin of viruses. The “virus-first” theory states that viruses predated the emergence of cells. At the opposite, the “reduction hypothesis” states that viruses evolved as reduced parasitic forms of early cellular organisms. The third one, “the escape hypothesis”, is a variation of the later stating that ancestral viral genomes were constituted of subsets of cellular genes that escaped cell control (reviewed in [35]).

I never personally understood how the first hypothesis could even be proposed, since it is properly absurd if we respect the precise meaning, accepted by all, of the word "virus": an obligate intracellular parasite. This mere definition immediately implies that the first virus(es) had to emerge in the context of preexisting cell-like organisms (free-living either as individualities or as parts of a consortium). The first viruses— in the sense that we give it today—could not precede the emergence of their hosts. Furthermore, the ancestor of the first virus(es) could not be one itself, but had to be an (or several) unknown free-living cell-like organism(s). From this point on, only some sort of reduction hypotheses should constitute the theoretical context on which to base the comparison of extant viral genomes and the reconstruction of their phylogeny.

**Figure 1:**
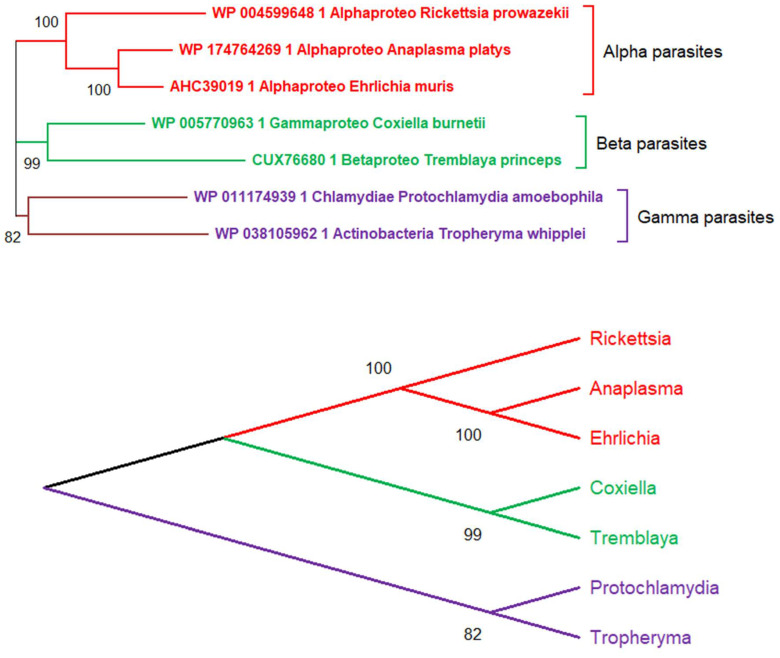
Erroneous phylogenetic relationships between seven obligate intracellular parasitic bacteria. **Top**: The neighbor-joining tree was generated from 1014 conserved sites in the multiple alignment of their DNA polymerase alpha subunits using the JTT substitution matrix. The protein NCBI identifiers are indicated. In absence of free-living bacterial relatives, the tree erroneously suggests (with a strong statistical support) the existence of 3 separate “parasite families” emerging from 3 distinct evolutionary branches (Alpha, Beta, Gamma). **Bottom**: Using a different representation, the tree topology (inherent to the tree-building algorithm) can be interpreted as supporting the existence of an ancestral obligate parasite from which all three families of extant parasites derived. The true evolutionary history of these parasitic bacteria is shown in Figure 2.

**Figure 2:**
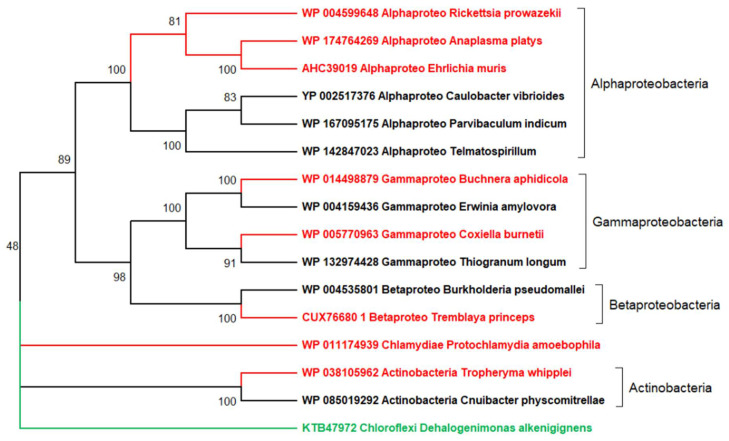
A more realistic representation of the origin and evolution of the obligate intracellular parasitic bacteria depicted in Figure 1. The neighbor-joining tree was generated from 974 conserved sites in the multiple alignment of 16 DNA polymerase alpha subunits using the JTT substitution matrix. The protein NCBI identifiers are indicated. The red branches correspond to the 7 obligate intracellular parasites while free-living relatives are in black. The green branch corresponds to a distant bacterium from the *Chloroflexi* phylum, used as outgroup. This tree suggests (with strong statistical support) that the parasitic bacteria independently originated at least 5 times from within 5 lineages also containing free living members: once from within *Actinobacteria* and *Betaproteobacteria*, twice from *Gammaproteobacteria*, and once early in the *Alphaproteobacteria* class from which three members of the order *Rickettsiales* emerged. In each case, the switch to a parasitic lifestyle was associated to the loss of essential genes (reductive evolution) nowadays documented by direct comparative genomics. One exception, visible in the tree, is *Protochlamydia amoebophila* for which no free living relative could be found. *P. amoebophila* belongs to *Chlamydiae*, a phylum of highly diverse members all of which have—like viruses—an obligate intracellular lifestyle. In the absence of known free-living relatives, the origin of this bacterial phylum remains mysterious. Compared to Figure 1, this figure illustrates how the lack of known free-living relatives might suggest totally erroneous evolutionary scenarios. The DNA polymerase was used as a conserved protein present in all bacteria (parasitic of not). Its viral version is frequently used in global phylogenetic reconstructions of eukaryotic dsDNA viruses.

There is, however, a fundamental difficulty in reconstructing the evolutionary history of obligate intracellular parasites by comparing them without reference to the free-living organisms from which they originated. For tree-based phylogenetic approaches to deliver a sensible scenario, all protagonists of the evolutionary game must be included in the analysis. This difficulty is illustrated in Figure 1 and Figure 2 where I attempted the phylogenetic reconstruction of 7 bacterial obligate intracellular parasites. Like viruses, those microorganisms cannot survive and multiply outside of eukaryotic cells which provide them with essential metabolites and enzymatic functions they no longer have. For the sake of my demonstration, I first pretended to reconstruct the phylogeny of these parasites, as if in search of the ancestral obligate intracellular parasite from which they might all have derived. Interestingly, these false premises resulted in a normal-looking tree, suggesting the existence of 3 different parasite “families” with strong statistical support (Figure 1, top). A different representation of this tree then suggests that these 3 families originated from a common “parasitic” ancestor (Figure 1, bottom), a convergence obviously imposed by all tree-building algorithms.

These conclusions are of course totally erroneous, as shown in Figure 2, where I incorporated free-living relatives to the analysis of the same seven parasitic bacteria. The resulting tree suggests an evolutionary scenario totally different from the previous one. The seven parasitic bacteria are now seen to relate to 5 different bacterial domains, 4 of which include a majority of free-living representatives (*Chlamydiae* being a noticeable exception). In contrast to Figure 1, this more realistic tree (further supported by a large body of genomic data) does invalidate the existence of an “ancestral” bacterial parasite from which all extant parasites would have derived. Numerous whole genome comparisons have demonstrated that obligate intracellular parasitic bacteria originated from their free-living relatives by the loss of essential genes and functions, an irreversible process of genome reduction through which they become increasingly dependent toward their hosts [37,38,39,40,55,56,57]. Interestingly, the 7 parasitic bacteria compared above (some of which encode close to 1000 proteins) share less than 100 “core” genes (involved in translation, DNA replication, and transcription), thus much less than the 400 or so genes considered to constitute a minimal free-living bacterial genome [58]. Without knowing the actual evolutionary history of these parasites (Figure 2), one might interpret these 100 core genes as those characteristic of their common parasitic lifestyle. This is of course wrong, as those genes were inherited from (and essential to) their free-living ancestors. On the other hand, these 100 core genes do not either constitute the entire genome of a hypothetical common ancestor (that had to possess a whole set of free-living functions), as it is most often concluded when similar phylogenetic reconstructions are applied to viruses. This then leads to the erroneous conclusion that obligate intracellular parasites (and thus viruses) evolved from simpler ancestors by acquiring genes instead of losing them [28,59].

In the context of the reduction hypothesis, the simulated analysis in Figure 1 parallels the protocol by which viruses are compared to investigate their evolutionary history. By definition, viruses do not have “free-living” relatives alongside which they could be compared. Without such reference, the deep reconstruction of the evolution of viruses seems unattainable and even conceptually flawed. Phylogenetic reconstruction must thus be limited to the family level, i.e., to group of viruses the ancestry of which can be traced back to a common quasi-extant virus by multiple genes and physiological criteria (a situation similar to the *Alphaparasites*/*Rickettsiales* in Figure 1 and Figure 2). For the same reasons explained above, the small number of core genes strictly shared by different eukaryotic dsDNA virus families (3 within phylum *Nucleocytoviricota*, zero within kingdom *Bamfordvirae*) [33] (Table 1) should not be interpreted as a characteristic of their putative common ancestor, rather as the expected result of a random succession of genes losses starting from an ancestral genome impossible to reconstruct, or as evidence of different origins altogether.

## 6. The Random Walk of Gene Losses: Another Main Hurdle in the Reconstruction of Viruses’ Evolution

As soon as an organism switches to the absolute parasitism lifestyle, the laws of neo-Darwinian selection, which apply to the usual conservative evolution of genes, change radically. The previously careful preservation of essential genes is replaced by the possibility of losing functions which can be subcontracted to the host. This trend is irreversible, and no obligate intracellular parasite has ever been documented to revert to a free-living lifestyle. “Once a parasite, always a parasite” appears to be one of the few absolutely respected mottos of microbial evolution [60]. As viruses are archetypes of (acellular) obligate intracellular parasites, I do believe that the irreversible succession of gene losses constitutes the dominant force in their evolution [2].

Given the central place held by the absence of the protein translation function in the definition of viruses [2,44], it is natural to postulate that the cascade of gene losses that led to the diversity of viruses we know today was initiated by that of an essential ribosomal protein (or rRNA). This would immediately make the defective microbe an “obligate intracellular parasite”, moreover confined in the cytoplasm of its host. This new environment, rich in metabolites of all kind as well as ATP could then be used as a rich culture medium for the emerging parasite. This would then open the door to further genome reduction by the losses of the redundant biosynthetic and bioenergetic pathways, until reaching the bare bones of the virus-encoded DNA transcription and replication apparatus. Alternatively, this process of retrogressive evolution may have been initiated within an already established cellular parasite becoming a virus via the loss of protein translation.

During this phase, the neo-Darwinian selection process will continuously select the viruses for an optimized parasitic lifestyle generating more progenies at each round of infection. However, this goal can be achieved in many different ways, sometimes contradictory, depending on the host and ecological situations. It could be via further genomic reduction (thus alleviating the energetic burden of DNA replication on the host) [61], by improving the efficiency of host infection (by innovating on virion structures and infection strategies) [12], by helping the host viability (thus increasing burst sizes) [62,63], or by using molecular defenses against viruses competing for the same host [64]. Such a complex web of evolutionary constraints is expected to generate a huge diversity of “optimal” solutions (sometimes involving moderate gene gains via horizontal transfers) resulting into the observed variety of viral gene contents without much apparent rationale.

If the loss of viral genes duplicating cytoplasmic functions (amino acids and nucleotide synthesis, energy metabolism, protein translation) probably can happen quickly, in an almost random manner (the virus benefiting from a free lunch within the cell), the loss of the functional virus-encoded DNA replication or transcription machineries must be concomitantly compensated by an access to the cellular ones, in the host nucleus. The viruses must thus evolve a strategy to either transport their genomes to the intact nucleus [42,65], or make it functionally “leaky” [23,66], or even dissolve it altogether [67]. The passage from a purely cytoplasmic replication cycle to a nuclear one is thus a major step in the continuous retrogressive evolution of viruses. I previously noticed that such transition appears to obey a strict order (loss of transcription first, then of replication). However, it seems to happen randomly at various stages of the genome reduction process, concerning viruses with vastly different genome sizes (Table 1).

**Figure 3:**
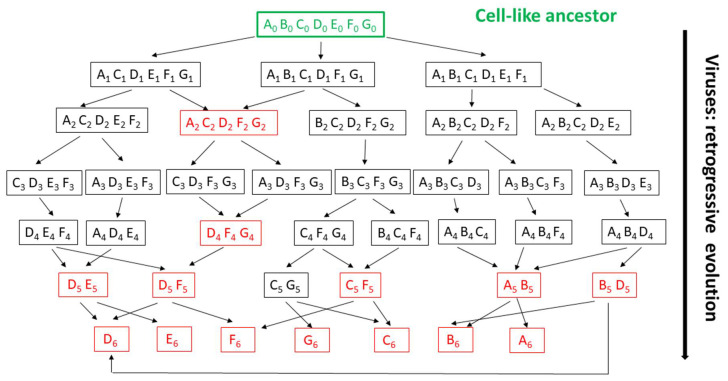
The “virus late” hypothesis: illustration of the intractable evolutionary scenarios resulting from random gene/function losses. A toy virus world is represented, starting from a hypothetical ancestral cell-like organism (level 0, w/o extant representative). Each box contains the abstract gene content inherited by a given virus (family) from its immediate ancestor. Red “genomes” indicate viruses with shared gene contents albeit possibly resulting from distinct evolutionary pathways. Random gene losses lead to very diverse overlaps of gene assortments (as in viruses) or to situations where no single “core gene” is shared by all virus family (here starting at level 3), as observed in actual viral genomes (in particular small ones). Individual genes recurring in multiple combinations (families) or ultimately remaining in the smallest genomes (level 6) are not more characteristic of the parasitic lifestyle than less ubiquitous ones. In addition, genes shared by more families than other (such as D) may not be better phylogenetic markers than others, as they could have been inherited from different ancestors (DE, DF, BD). Their polyphyly will not be detected if some of their above ancestors are extinct or unknown. This graph illustrates the difficulty of reconstructing the deep phylogeny of viruses beyond the immediate family level both due to the capacity of random gene losses enjoyed by obligate intracellular parasites and the lack of associated free-living organisms to be used as references.

To illustrate two main points, a diagram of virus evolution through genome reduction from a cell-like (non-virus) ancestor is represented in Figure 3. First, soon after a parasitic lifestyle is initiated, random gene losses generate very different genomes, from which common-to-all genes (the so-called “core” genes) have no reason to be maintained and could disappear rapidly. This corresponds to reality, where the discovery of new virus families steadily led to the reduction of the number of core genes [12,29,33,51,66,67,68,69,70]. However, while other authors attribute this phenomenon to the “high rate of horizontal transfer and fast sequence divergence of virus evolution” [71], it is in fact intrinsic to the reduction hypothesis. “Core” genes are borne to disappear as our knowledge of the viral diversity increases because the concept of virus simply does not imply the existence/conservation of any specific virus-encoded function. Core genes are not intrinsically “essential” to viruses, but are just the artefactual (provisional) consequence of our finite and incomplete samplings of the virosphere.

Second, even when similar genotypes (i.e., assortment of core genes) are recognized in different viruses they cannot be used as reliable evidence of common ancestry as they could originate from totally unrelated evolutionary pathways from which intermediate viral forms have disappeared or are not yet discovered. In absence of such intermediates, polyphyletic viral clades might erroneously appear monophyletic (eventually promoting irrelevant taxonomic clustering). Such cases are illustrated by the genotypes indicated in red in Figure 3. Thus, the more ubiquitous a core (or quasi-core) gene appears to be, the more likely are the virus groups exhibiting it to be polyphyletic (see gene D as an example, Figure 3). One then expects phylogenetic trees built from the most shared genes to be highly discordant. This has been a common finding [29,30,33,51,52,69,70], prompting authors to abandon tree-based phylogenetic reconstruction methods for network representations [68,71], to extrapolate homologies from non-significant sequence similarity [72], to invoke unwarranted combinations of losses and horizontal gene transfers [33,70,73], to abandon the grail of a unique common ancestry [73], or to ignore whole virus families causing troubles [33].

## 7. Conclusions

In this conceptual article, I showed that the phylogenetic reconstruction of the evolution of viruses suffers from several fundamental limitations, in the hypothesis that they were derived from cell-like microorganisms, the only logically sound scenario if we respect the definition of viruses as “obligate intracellular parasites”. One limitation is due to the lack of free-living lineage(s) against which to compare the various virus families (Figure 1). The other is due to the almost complete relaxation of functional constraints which characterizes a microorganism having switched to an obligate intracellular parasitic lifestyle. Paradoxically, the evolutionary trajectory of a virus is much better defined by the way it has lost genes, rather than by the nature of those it has kept. Unfortunately, one can only compare viral genomes on the basis of the later.

In this paper, I voluntarily neglected two additional confounding evolutionary processes: (i) the acquisition of genes by horizontal transfers from cells or other viruses, (ii) the de novo creation of genes by the viruses themselves [74]. It is nevertheless clear that these two processes could only make phylogenetic reconstruction even more intractable.

I made no hypothesis on the very nature of the ancestral microorganism(s) at the origin of viruses, but it was in all likelihood equipped with a DNA transcription/replication machinery, and protein synthesis. I proposed that the loss of the later is the evolutionary event that initiated all dsDNA virus lineages, for which there is no evidence—or logical need—that it only happened once.

Viruses are traditionally classified into families on the basis of common infection and intracellular replication strategies, overall particle structures, and large, specifically shared gene contents attesting their descent from a common (quasi-extant) viral ancestor. Cladistics (i.e., the presence/absence comparison of entire gene contents) is often a convenient and sufficient method to delineate families that should clearly appear as monophyletic clades (e.g., in [74]). It is nevertheless not a fool proof exercise as illustrated by Figure 1 and Figure 2. The recent reclassification of certain members of the *Phycodnaviridae* family into that of *Mimiviridae* [10] (which could not have been possible prior to the discovery of Mimivirus and its relatives [6]) is a good illustration of the risk of freezing a taxonomic classification too early.

Having listed the fundamental limitations plaguing the deep phylogenetic reconstruction of viruses beyond the family level, I can only wonder about the merits of a spectacular fifteen-rank classification hierarchy recently adopted by the ICTV [41]. Families are now aggregated in a succession of 10 taxonomic levels (suborder, order, subclass, class, subphylum, phylum, etc.), the monophyly of which, according to the argument presented here, may remain forever beyond the realm of scientific evidence. In addition to being nearly impossible to pronounce or memorize, many of these abstract clades are dangerously suggesting totally unsupported related ancestries between families as different as *Mimiviridae*, *Adenoviridae*, *Phaeovirus*, and virophages (all included in the *Bamfordvirae* kingdom), or the *Herpesviridae* and a large number of phages (now included in the *Heunggongvirae* kingdom). While I fear that this new taxonomical scheme will be taken as a word of the gospel by the incoming generation of virologists, I am also confident that the future discovery and characterization of many more unconventional viruses will quickly convince them that any attempt to lock viruses into such a deep and rigid classification does not make any biological sense.

## Figures and Tables

**Table 1:** Virus-encoded DNA/RNA polymerases in various eukaryotic dsDNA viruses.

Family/Genus Name	DNA Polymerase	RNA Polymerase	Genome Size Range
**In kingdom *Bamfordvirae***			
*Mimiviridae*	+	+	0.4–1.6 Mb
*Poxviridae*	+	+	185–360 kb
*Iridoviridae*	+	+	100–212 kb
*Asfarviridae*	+	+	171–190 kb
*Ascoviridae*	+	+	120–200 kb
*Coccolithovirus* ^1^	+	+/-	407 kb
*Marseilleviridae* ^1^	+	+/-	350–376 kb
*Chlorovirus*	+	-	280–300 kb
*Prasinovirus*	+	-	173–199 kb
*Adenoviridae*	+	-	25–45 kb
*Lavidaviridae*	-	-	17–30 kb
**In other kingdoms or unclassified**			
*Pithoviridae*	+	+	610 kb
*Pandoraviridae*	+	-	1.8–2.5 Mb
*Nimaviridae*	+	-	309 kb
*Herpesviridae*	+	-	108–236 kb
*Nudiviridae*	+	-	97–232 kb
*Baculoviridae*	+	-	80–160 kb
*Polydnaviridae*	-	-	up to 800 kb
*Papillomaviridae*	-	-	7 kb
*Polyomaviridae*	-	-	4–5 kb

^1^ The virus-encoded DNA-dependent RNA polymerase is not packaged in the virion.
